# Local topological features of robust supply networks

**DOI:** 10.1007/s41109-022-00470-2

**Published:** 2022-05-20

**Authors:** Alexey Lyutov, Yilmaz Uygun, Marc-Thorsten Hütt

**Affiliations:** 1 Department of Mathematics and Logistics, Jacobs University Bremen, Campus Ring 1, 28759 Bremen, Germany; 2 Department of Life Sciences and Chemistry, Jacobs University Bremen, Campus Ring 1, 28759 Bremen, Germany

**Keywords:** Network motifs, Minimal model, Supply chain management, Spatial networks

## Abstract

The design of robust supply and distribution systems is one of the fundamental challenges at the interface of network science and logistics. Given the multitude of performance criteria, real-world constraints, and external influences acting upon such a system, even formulating an appropriate research question to address this topic is non-trivial. Here we present an abstraction of a supply and distribution system leading to a minimal model, which only retains stylized facts of the systemic function and, in this way, allows us to investigate the generic properties of robust supply networks. On this level of abstraction, a supply and distribution system is the strategic use of transportation to eliminate mismatches between production patterns (i.e., the amounts of goods produced at each production site of a company) and demand patterns (i.e., the amount of goods consumed at each location). When creating networks based on this paradigm and furthermore requiring the robustness of the system with respect to the loss of transportation routes (edge of the network) we see that robust networks are built from specific sets of subgraphs, while vulnerable networks display a markedly different subgraph composition. Our findings confirm a long-standing hypothesis in the field of network science, namely, that network *motifs*—statistically over-represented small subgraphs—are informative about the robust functioning of a network. Also, our findings offer a blueprint for enhancing the robustness of real-world supply and distribution systems.

**Supplementary Information:**

The online version contains supplementary material available at 10.1007/s41109-022-00470-2.

## Introduction

The analysis of network motifs (Shen-Orr et al. 2002; Milo et al. 2004; Alon 2007) goes back to the early phase of network science (Strogatz 2001; Albert and Barabási 2002; Barabási 2016). In contrast to studying the large-scale topological features of complex networks (e.g., their broad degree distribution or their hierarchical organization) or the microscale of properties of individual nodes (e.g., the betweenness centrality or the local clustering coefficient), network motifs have drawn the attention to a ’mesoscale’^1^ with the hope of explaining some of the functional properties of complex networks via the networks’ non-random features on this scale of organization.

In fact, *motif signatures*—patterns of non-random occurrences of certain few-node subgraphs—have been identified and associated to the networks’ functional categories (Milo et al. 2004) and have been, in subsequent studies, linked to the robustness of the networks’ dynamical function, e.g., for Boolean dynamics (Klemm and Bornholdt 2005) and flow networks evolved towards a robust performance under random deletion of links or nodes (Kaluza et al. 2007; Kaluza and Mikhailov 2007; Kaluza et al. 2008; Beber et al. 2013).

The question addressed in our investigation is whether the deep relationship between motif signatures and robust functioning identified in network science translates to supply networks as well.

Supply networks are shaped by a multitude of factors, including technological constraints, human preferences, available infrastructures and earlier relationships among suppliers and producers.

Modeling is often used to ask for the effect one of the influencing factors might have on supply networks, when considering this factor isolated from all other factors. Network formation games (Bloch and Jackson 2006; Fiat et al. 2006; Chekuri et al. 2007; Anshelevich et al. 2008), for example, focus on human preferences and decision patterns and embed supply networks in a game-theoretical framework. The structural consequences of resilience (Li et al. 2020) [see also the systematic literature review in Aldrighetti et al. (2021)] and ’network health’ (Basole and Bellamy 2012, 2014) are other topics often addressed via mathematical modeling of supply systems.

Other modeling approaches of supply networks include fuzzy programming (Fazlollahtabar et al. 2013), partial differential equations (D’Apice et al. 2009) and agent-based modeling (Li et al. 2020).

Here we ask, what network structures emerge, if robustness of the network is an important criterion. Our model is in the long tradition of minimal models (or ’toy models’) often employed in econophysics and other application domains of statistical physics (Kutner et al. 2019), in contrast to parameter-rich computational models. Such minimal models have the goal of understanding the ‘stylized facts’ (Buchanan 2012) of such real-world complex systems.

For supply networks robust functioning is of utmost importance. Key aspects in the infrastructure of our industrialized world depend on it. As a consequence, the topic of supply network robustness has received substantial scientific attention. Methods from nonlinear dynamics have been applied to study supply and distribution networks under fluctuations (e.g. Ritterskamp et al. 2018; Demirel et al. 2019). Using methods of nonlinear dynamics, especially parameterizing fixed points together with a stochastic sampling of the unknown entries of the Jacobi matrix (generalized modeling, Gross and Feudel 2006) were analyzed in Ritterskamp et al. (2018) and Demirel et al. (2019) (see also Gross et al. 2018).

Typical approaches to quantify the robustness is to jointly consider functional and structural aspects (Dong 2006) or to view robustness as a function of declining service level under random or targeted attacks (Adenso-Díaz et al. 2018). Some works analyze real-world networks using the robustness metrics (Brintrup et al. 2016; Zhao et al. 2019). Only few investigations design or simulate networks based on a robustness criterion (see also the literature review on robustness, responsiveness and resilience provided by Klibi et al. (2010)). Exceptions include work on simplified supplier-retailer (Wei et al. 2015) or agent-based (Nair and Vidal 2011) models and simulate, design and optimize supply networks using the robustness criterion. Network motifs or similar topological features as indicators of robustness have not been discussed in the supply network literature.

At the same time, supply and distribution networks are high-dimensional systems with high demands on efficient organization and the fulfillment of logistic target values. The design and operation of such networks are usually performed based on both local and global information (Blunck et al. 2018) and under the influence of other supply networks (Matous and Todo 2017). These aspects are often addressed by optimization methods (e.g. Hendriks et al. 2012; Garcia and You 2015). In Hendriks et al. (2012); Armbruster et al. (2011), an abstract formulation of logistic networks (supply and distribution networks) has been formulated as an optimization problem.

Methods from network science have been particularly employed to supply systems to analyze the impact of disruptions, such as transportation failure or supply shortages, and hence the robustness and resilience of such systems (e.g. Helbing et al. 2004; Sun and Wu 2005; Atalay et al. 2011; Brintrup and Ledwoch 2018; Arora and Ventresca 2018; Perera et al. 2017). In Atalay et al. (2011), the value of network representations of supply networks for an understanding of economic processes was elaborated, with an application focus on the automotive industry.

The resilience and vulnerability of supply chains and supply networks to disruption were analyzed—particularly in light of the COVID-19 pandemic—in Ivanov and Dolgui (2020) and Ivanov (2020). An overview of the important field of mathematical modeling of sustainable supply chains is provided by Seuring (2013).

The embedding of supply networks in real geographical space, the often multi-modal nature of supply networks (distributing not a single good or material, but rather a whole range of goods and materials, which are often interdependent), as well as the weighted nature of supply networks (where suitable weights of edges are the total *volume* shipped along this edge in a certain time window, or the total *value* or the average *cost* per shipment, which in turn is partly related to geographical distance) all make formal network representations suitable for the analysis, e.g., of network motifs, challenging.

In order to account for these incompatibilities between abstract network representations and real-world features of these systems, we introduce a stylized supply network model, which retains the spatial embedding and the overall ’source-to-target’ organization of supply networks, but is generic enough to allow for a motif analysis of the resulting networks.

## Methods

### Supply network model

**Fig. 1:**
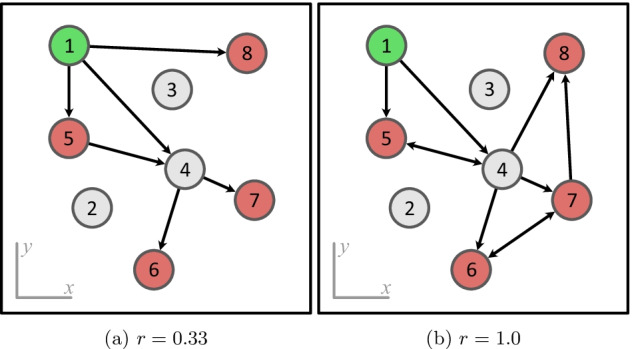
Examples of model setup and possible networks with a low (**a**) and high (**b**) robustness. Values of $r$ are indicated below each network. In (**a**), $E_{r} = {(1,4), (5,4)}$, M=6. In (**b**), any edge can be removed without reducing the demand stisfaction, $|E_{r}| = M = 10$

Our supply network model consists of *N* nodes that are spatially distributed on a 2D plane (Fig. 1). Each node can have one of three roles: producers (green in Fig. 1) that are generating a product, demanders (red) that require the product to be delivered, and intermediate nodes (gray) that neither produce, nor demand the product, but can be used to deliver the product efficiently (warehouses). A model setup is a set of *N* nodes, each with (*x*, *y*) coordinates and an assigned role. All setups used in the experiments in this paper have only one producing node and $N_{d}=N/2$ demanders. In the scenario of a single-product systems, adding more producers results in much simpler networks that have little variation in network structures. In the current research, we focus on single-product single-producer systems. The coordinates of nodes for a single setup are sampled from a random uniform distribution.

**Fig. 2:**
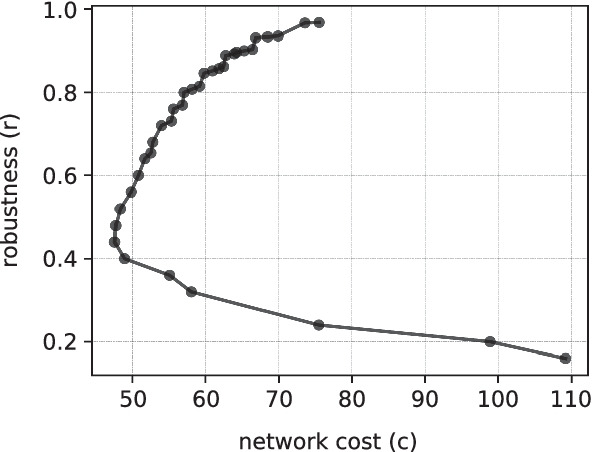
An example of a typical Pareto front of an ($c$, $r$) optimization

A supply network is a set of *M* directed edges that represent transportation routes in the system. Edges can start and end at any type of node: Cases, where an edge goes to producer or warehouse, represent resupply of delivery vehicles. Cases, where an edge goes from a demander node, represent parts of subsequent delivery routes. Each network has the following parameters: number of edges *M*, network cost $c$, and robustness $r$. Network cost $c$ is the sum of the Euclidean distances of all edges in the network. It reflects how optimal the product paths in the network are with respect to edge length. Robustness is a metric that shows how susceptible the network is to a random loss of edges. It is computed by finding the subset of edges $E_{r}$ such that any edge from $E_{r}$ can be safely removed from the initial network and the resulting network will still have paths from a producer to every demander. The robustness $r$, in this case, is $r=|E_{r}|/M$. All networks in this research were required to have a full demand satisfaction.

A focus of our investigation is the analysis of these supply networks from the perspective of few-node subgraphs. To this end, we follow the concept of a *motif signature* proposed by Milo et al. (2004). A motif analysis shows how over- or underrepresented certain 3-node subgraphs are in the investigated network. The analysis is done by comparing the frequency of each of 13 possible subgraphs (see Additional file 1: Fig. S1) in the original network to its randomized versions via a normalized vector of z-scores. In other words, we count every 3-node subgraph in the original network ($x_{i}$), perform multiple randomizations of the network by switch-reconnecting the edges, count the subgraphs in the randomized networks, and compare the count in the original network to the count distributions of the randomized networks (mean $\mu _{i}$, standard deviation $\sigma _{i}$). Components of the resulting vector are: $z_{i} = (x_{i} - \mu _{i}) / (\sigma _{i} S)$, $i=1, \ldots , 13$, where $S=\sqrt{\sum _{n=1}^{13} ((x_{i} - \mu _{i}) / \sigma _{i})^2}$. High z-scores of certain subgraphs—feedforward loop and bidirectional feedforward loop (7 and 9 in Additional file 1: Fig. S1) are associated with high robustness because they provide alternate routes using few links. Simpler subgraphs (1–5 in Additional file 1: Fig. S1) are usually associated with low robustness but high responsiveness of the network, as they allow to distribute materials or information quickly, but lack alternative routes. Another notable subgraph is the feedback loop (8 in Additional file 1: Fig. S1).

**Fig. 3:**
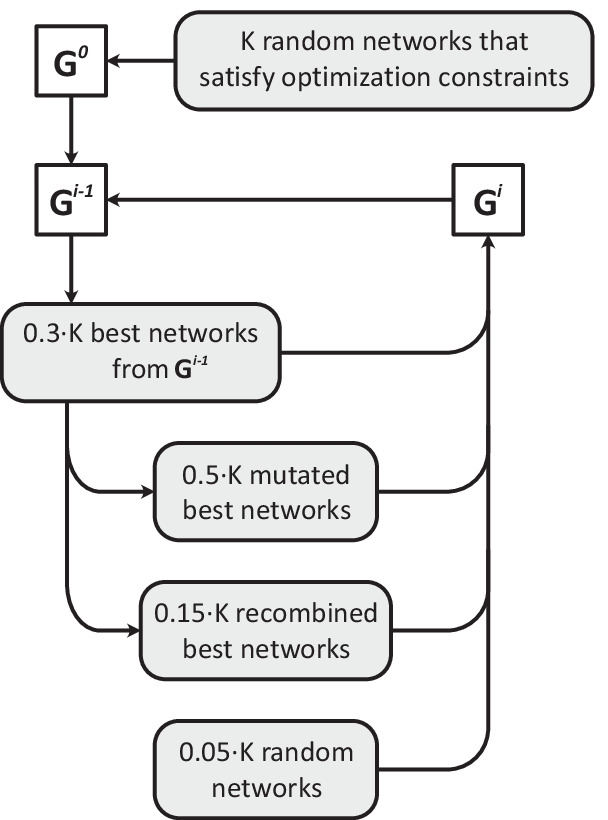
Scheme of the optimization process. First, $G^{0}$ is created as random networks that satisfy the optimization constraints (*M*, edge length, etc.). Then, to create the new generation, the algorithm picks the best networks from the previous generation and creates new networks by mutating and recombining the best networks. The new generation is finally created by combining the best networks from the previous generation, mutations and recombinations of randomly selected best networks, and purely random networks that satisfy the optimization constraints. Then, the procedure is applied to the new generation. The process is repeated for $G^{N}$ generations

**Fig. 4:**
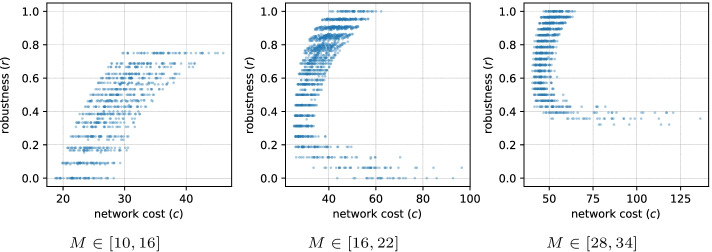
Pareto fronts in a series of $(c, r)$ optimizations with $N=20$ and varying *M* boundaries. Each figure combines the results of 50 model setup runs that have different spatial distribution of nodes

Randomized networks in motif analysis can be generated using different versions of the null model. Here we have used the default setup used in the original research (Milo et al. 2004), the version that does not preserve the mutual edges, and our custom null model that generates networks with the same level of demand satisfaction as the original network. To make the results more comparable, the default null model that preserves mutual edges has been used in the main part of our investigation, while the examples of results for the other null model variants are shown as Additional information. In general, the association of robustness with a non-random subgraph composition is observed in all null model variants. Motif calculations were performed using the mfinder software developed by Alon et al. (2002).

### Numerical simulations

To solve a given setup of the model, it is necessary to find a network that connects producers and demanders in a robust and cost-efficient way. The problem of generating robust networks is computationally complex and has no analytical solution or simple yet efficient heuristic. It can be formulated as a multi-objective optimization problem with objective functions $min(c)$, $max(r)$, and additional constraints, e.g. the number of edges *M* or constraints on the edge lengths. The optimization problem is solved using genetic algorithms (Deb et al. 2002) with a small modification that allows simultaneous maximization and minimization of a target objective. This modification might be necessary in optimization problems where the allowed number of edges in a network has a lower boundary $M_0 \le M$. The shape of the Pareto front in this case might have two parts: below and above the minimal network cost (see example in Fig. 2). In this case, for the networks that have robustness lower than the robustness of the network with the best $c$ ($r<0.44$ in the figure), the problem of minimizing the robustness is solved. For the networks above, the robustness is maximized. The critical robustness is re-evaluated after each generation, as the front evolves.

**Fig. 5:**
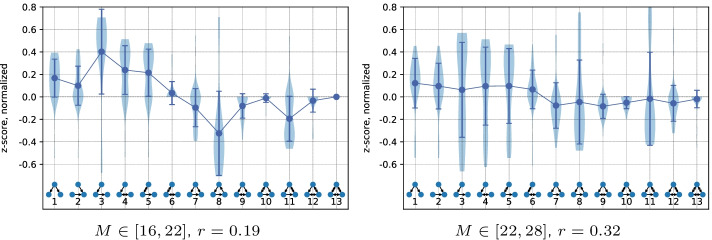
We generate 50 different network setups with $N=20$ and for each setup perform a $(c, r)$ optimization with restriction on the allowed number of edges *M* (shown below each figure). From the resulting Pareto fronts, we take 50 vulnerable networks with given $r$ and compute their motifs. For a single network, the result of motif computations are 13 z-scores that indicate how over- or underrepresented each subgraph is in the original network, compared to its randomized versions. This gives 50 z-score values for each of the subgraphs that form a distribution drawn with shaded vertical violin plots. Blue circles in the figures are the mean values and vertical lines with ticks are the standard deviations of these distributions

**Fig. 6:**
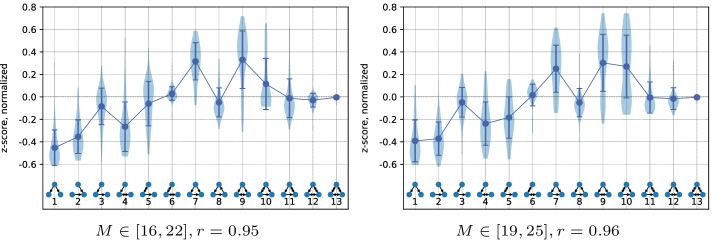
Motif patterns of robust networks in $c$, $r$ optimization with $N=20$. Similarly to Fig. 5 we take 50 networks with high $r$ from the Pareto fronts

During the optimization loop starts with picking the best networks from the previous generation $G^{i-1}$ based on their rank (number of networks that are better at least by 1 optimization criterion). Then, new networks are created by mutating and recombining random best networks. There are three possible mutation procedures—removing, adding, or replacing random edges. The recombination procedure takes two networks, selects a random subset of edges from each, and outputs the network with the union of the selected edges. The new generation $G^{i}$ is finally created by combining the best networks from the previous generation, mutations and recombinations of randomly selected best networks, and purely random networks that satisfy the optimization constraints. The first generation $G^{0}$ is created as random networks that satisfy the optimization constraints (*M*, edge length, etc.). The process is repeated for $G^{N}$ generations without an explicit convergence stop. A schematic diagram of the optimization procedure is given in Fig. 3. Each optimization starts with its own random networks, ensuring that optimization runs for different model setups (different *N*, *M*, or node locations) do not affect the results of each other.

**Fig. 7:**
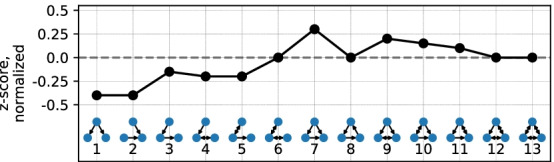
The target network motif pattern typical to the robust biological systems, reproduced from Milo et al. (2004). X axis represents different subgraphs, Y axis shows normalized z-score of the corresponding subgraph

**Fig. 8:**
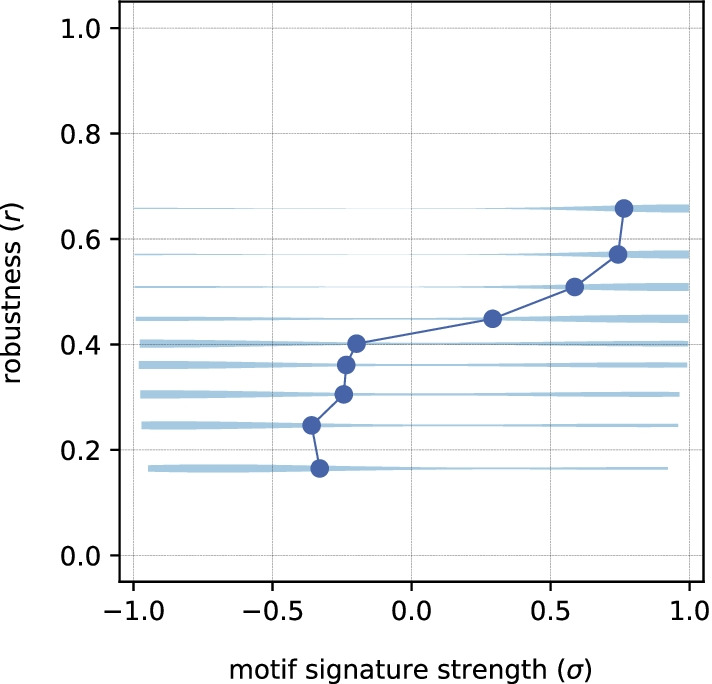
Distribution of $r$ values as a function of $\sigma$ for the results of $(c, \sigma )$ optimization. Sets of networks that correspond to given robustness are plotted as violin plots to demonstrate their distributions, while the solid line shows the behavior of their means

## Results

### Robust supply networks

In the first set of numerical experiments, we investigated how the robustness shapes supply networks on a structural level. To do so, we optimize robustness $r$ and network cost $c$ for 50 setups with the different spatial distribution of nodes while keeping the node roles fixed. For each network setup, the optimization algorithm starts with its own random networks and runs independently. In the result, for each single setup there is a Pareto front of several networks, each with its own $c$ and $r$. Then, from each front we take one network with a corresponding $r$ and analyze them together. During the whole process, different setups do not interact with each other, networks are formed independently and cannot affect or bias the networks in other runs.

It is clear that the allowed number of edges in a network *M* has a direct impact on the robustness. It is much easier to install robustness with more edges, as the number of alternative paths increases rapidly with higher connectivity, e.g. by having direct edges from the producing node and duplicating them through one intermediate node. On the other hand, when *M* is close to the minimal number of edges required for full demand satisfaction, no edges can be used as alternative paths. To make networks in both cases more specific, we have defined the minimum $M_{min}$ and the maximum $M_{max}$ number of edges allowed in the optimal networks and varied these boundaries, solving 50 different setups for each pair ($M_{min}$, $M_{max}$).

The first series of optimizations are performed for $N=20$ nodes with $(M_{min}, M_{max}) \in [(10, 16), (13, 19), \ldots ,(40, 46)]$. Inspecting the optimization results in form of Pareto fronts (Fig. 4) it can be seen that depending on the allowed number of edges high or low robustness areas become less populated. To analyze the structures of robust and vulnerable networks, we are sampling one network with low and one network with high robustness from each of the 50 optimizations. The low and high robustness, in this case, are defined as 10 and 90 percentiles of the $r$ values for each edge limit.

**Fig. 9:**
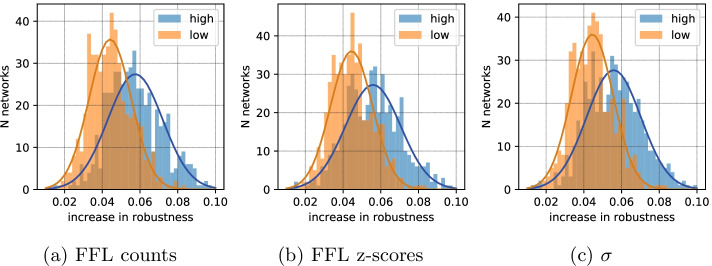
Histograms of the average increase in robustness for high and low changes in one topological metric. We take 500 random networks that have full demand satisfaction and some robustness. For each combination of a network and an edge that is not in the network, we compute *r*, motif signature, and three-node subgraph counts. Then, for each network, we separate combinations into two groups: the ones that yield the highest increase in a simple metric (counts of feedforward loop (**a**), z-score of feedforward loop (**b**), and $\sigma$ (**c**))—denoted as high, and the remaining combinations—denoted as low. After this we compare the average increase in *r* in these two groups

**Fig. 10:**
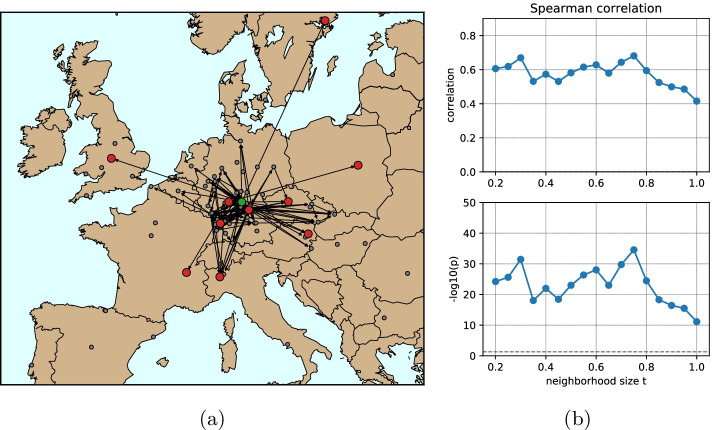
Application of the model to industrial data. **a** shows a subnetwork of the full European supply network of an automotive company. The subnetwork is generated by selecting the routes used for transporting one product category and taking a neigborhood of these routes with size parameter $t=0.6$. In **b** all product subnetworks are analysed together using Spearman correlation (upper panel) and the corresponding p-value (represented as $-\text{ log }(p)$; lower panel) of their $r$ and $\sigma$ values for different neighborhood sizes *t*. The dashed lines indicate zero correlation (upper panel) and $-\text{ log }(0.05)$ (lower panel), respectively

Figures 5 and 6 show motif patterns of low and high robustness networks from 50 different setups. The analysis of different *M* boundaries shows that the range between (16, 22) and (19, 25) yields the clearest signal for both vulnerable and robust networks. The vulnerable networks have a pattern similar to the superfamily associated with words sequences in languages from Milo et al. (2004) or the node-robust networks in Kaluza et al. (2007). Simpler subgraphs (1–5) in such networks are overrepresented, while the more complex subgraphs that contribute more to the robustness (7, 8, 9, 11) are underrepresented. The main difference with the languages superfamily in the vulnerable networks is the role of the feedforward and feedback loops. The lack of feedforward loops is much less significant, while the underrepresentation of the feedback loops is the feature that distinguishes the vulnerable supply networks from the null model. As can be seen in Fig. 5, the signal of vulnerable networks disappears fast with the growth of *M*, as the low robustness becomes hard to achieve with more edges.

The robust networks have an over- and underrepresentation pattern of three-node subgraphs similar to the second superfamily from Milo et al. (2004). This superfamily is associated with biological networks (signaling network of living organisms, gene regulatory networks, neuronal networks). Important features of this motif signature are the neutrality of z-scores for subgraphs 3, 6, 8, the importance of the feedforward loop and its bi-directional version (7, 9). This pattern is also present across a wider range of *M* compared to the signature of vulnerable networks. The signal, however, becomes less informative at the higher *M* values, as installing robustness becomes easier with the help of any of the subgraphs. This appearance and decay of both high and low robustness signals can be observed in Additional file 1: Fig. S2, where the pattern strength is plotted as the function of robustness $r$. Around $M \in [16,22]$ and [19, 25] the signal is the strongest, showing the biggest difference between high and low robustness networks. Networks with the lowest $M \in [10,16]$ and [13, 19] have too few edges to reach the peak of signal: on the one hand, the highest robustness of $r> 0.8$ is hard to achieve, on the other hand, vulnerable networks produce degenerate motif patterns with only few subgraphs present.

Similar experiments on smaller ($N=10$, Additional file 1: Fig. S3) and larger ($N=30$, Additional file 1: Fig. S4) networks show the same motif signatures for both robust and vulnerable networks. The peak strength of the pattern signal appears when $M \approx N$, indicating that for the investigated setup the signal strength depends on the network’s average degree, rather than on the connectivity.

In order to further investigate the similarity of the motif signature obtained here with the corresponding superfamily from Milo et al. (2004), we compute the Pearson correlation coefficient of motif signature in our model with the representation of the superfamily signature shown in Fig. 7. We denote this correlation coefficient the signature strength $\sigma$ and the superfamily signature as *target z-score vector*. This quantity will be analyzed in detail in the following section. The resulting network parameter $\sigma$ shows how close the motif pattern of the network is to the target pattern. The value of $\sigma$ can vary between $-1$ and 1.

### Motifs as a way to generate robust networks

In the first part of our investigation, we have seen how the robust networks tend to have a rather precise set of local topological features. In other words, the majority of the robust networks lie in the set of networks with a particular motif signature which, in fact, has already been associated with robustness in previous studies, in the context of regulatory systems (Milo et al. 2004) and layered flow systems (Kaluza et al. 2007). As a next step we will now address the opposite direction of this statistical association between robustness and motif signatures: If we generate a network with the required pattern, will it necessarily be more robust than the others? Or will such a network have the same high level of robustness but at a lower network cost? In the existing literature, an approach of installing robustness via generating a certain motif pattern is not well investigated, perhaps related to the computational complexity of computing network motifs, which is $O(M^4)$, as compared to the $O(M^2)$ for the robustness. This is a relevant question, as in principle it could be that the set of networks with the pattern is bigger than the set of the robust networks and it is possible to generate a vulnerable network with the given pattern.

In order to address this question, we perform a full optimization for the given motif pattern. Furthermore, we suggest a heuristic that indirectly installs the pattern, but does that with a smaller computational complexity.

#### Full motif pattern optimization

Similar to the robustness optimization, we solve an optimization problem for 50 different setups with the random spatial distribution of nodes. The objective functions in this case are the network cost $c$ and the signature strength $\sigma$, i.e. the correlation of the network’s z-score vector with the target vector given in Fig. 7. Due to the time complexity of optimizing $\sigma$, this experiment has been performed for a single set of edge boundaries $(M_{min}, M_{max}) = (16, 22)$ and $N=20$, i.e. the edge range for which the low and the high robustness signals were the strongest. This optimization had one additional constraint, namely that only edges not longer than a certain length *l* were allowed to form networks. The allowed length *l* is defined as 120% of the length that makes the network connected. Our numerical simulations have shown that this length constraint greatly enhances the association of the motif signature with robustness. Regarding the impact of edge length, and thus spatial locality of the network, on the motif patterns one can refer to Artzy-Randrup et al. (2004).

From the Pareto front of this optimization (Additional file 1: Fig. S5) it can be seen that, although the generated networks have reached both extremely high and low $\sigma$ values, the robustness range is not entirely covered, as opposed to the $c$, $r$ optimization (Fig. 4). Even more striking, networks with the opposite motif pattern (negative $\sigma$ values in Additional file 1: Fig. S5) reach robustness values that are comparable to those with target pattern. This observation indicates that not all networks with the given motif pattern will necessarily be robust. On the other hand, looking at the robustness as the function of motif patterns (Fig. 8), it can be seen that high robustness networks have almost exclusively the target motif pattern. Looking at the motif z-scores of high and low robustness networks in this optimization (Additional file 1: Fig. S6), we find patterns similar to those from $c$, $r$ optimization. For higher robustness, there is an overrepresentation of subgraphs 7, 9 and 10 with underrepresentation of subgraphs 1–5. For lower robustness, there is an overrepresentation of the first 5 subgraphs and underrepresentation of subgraphs 7–11.

#### Heuristic motif pattern enhancement

The approach with direct motif pattern optimization has proven to be both computationally complex and inefficient in generating robust networks. Here we try to address these problems by testing a heuristic that follows simple rules based on motif patterns. In this approach, we consider a more practical setup, with a randomly given network that has a full demand satisfaction. The goal is to insert one edge into the network such that the insertion brings the existing motif signature closer towards the target signature, increasing the robustness in the process.

We investigate this problem by taking 500 different random networks with $N=20, M \in [16, 22]$ and inserting every edge that did not exist in the original network. The extended networks are then compared with the basic one by three parameters: count of feedforward loops (*c*07), z-score of the feedforward loop (*z*07), and overall correlation with the target z-score pattern ($\sigma$). After that, we split all the possible networks into two groups: **high**—those that give the maximum increase in *c*07, *z*07, and $\sigma$ and low—the remaining networks. In the case of *c*07, the high group is composed of networks with positive change in the 7th subgraph counts. In the case of *z*07 and $\sigma$, the top 10% of the networks are taken (see more details in the additional file information, Additional file 1: Fig. S7). Then we compare the increase in robustness in these two groups taking their mean values. This process is then repeated 500 times for different base networks and the histogram of high and low means is finally plotted (Fig. 9).

The resulting figure tells us that all three parameters work equally well for edge insertion. Selecting the edges with the highest values of *c*07, *z*07, or $\sigma$ leads to a higher increase in robustness. To our knowledge, these two investigation steps—the full motif pattern optimization and the heuristic motif pattern enhancement—are the first examples indicating that indeed this motif signature *implies* robustness. However, in both numerical experiments, the observed effect is weak. Furthermore, even the heuristic is still more computationally complex than the direct robustness computations, even using the simplest *c*07 approach. These observations show that the results, though of relevance for the theoretical understanding of supply network robustness, will most likely not be of immediate practical relevance. One possible application of such a heuristic might be in the case when computing or defining robustness is hard.

### Application to industrial data

Next, we apply the concept of the model to a real-world supply network. As an example, we explore the transportation network of all European facilities of a global automotive supplier. These facilities both produce and demand products and the whole network is an overlay of a large product portfolio. The company organizes internal deliveries on its own meaning there is no competition among facilities and products. However, for most of the product categories, there is more than one producer and transportation links might be used to deliver several product categories. To bring this aspect closer to the scenario investigated in the model, we consider each of the 627 product categories independently and extract subnetworks that are used to deliver only the selected product. We also consider only one producer for each product category—the one that has the largest produced volume.

With these assumptions, the resulting data contains multiple single-producer transportation subnetworks that are used to distribute one product category. Such networks predominantly consist of direct routes from the producer to demanders and have zero robustness because every route is vital for the demand satisfaction. In reality, as the whole transportation network consists of multiple products and deliveries are combined together, the actual transportation network for a single product includes additional routes. To model this behavior, we introduce an additional local neighborhood size parameter *t*. This parameter represents the number of additional routes from the whole transportation network data that are included in the transportation network of a single product. When $t=0$, the transportation network consists purely of direct links from the producer to demanders. With highest *t*, the network turns into the full transportation network from the data. For the intermediate values, the network includes the direct single-product routes plus those indirect routes that do not exceed the length of the direct route times *t* (see an example of a product subnetwork in Fig. 10a).

The the result, we vary the parameter *t* thus getting product subnetworks of different size and analyze the parameters $r$, $\sigma$ of the resulting networks. As expected, for higher values of *t* the robustness also increases. More interesting is whether for a given *t* there is a connection between $r$ and $\sigma$. A Spearman correlation analysis of these two quantities in Fig. 10b, shows a strong $(> 0.5)$ and significant $(p-value<< 0.05)$ correlation of these two quantities. Analysis of the Pearson correlation coefficient confirms these observations (see Additional file 1: Fig. S8) but is less reliable due to the non-Gaussian distributions of the quantities investigated. As we are going to the region of bigger neighborhood sizes, product sub-networks include more and more edges and become closer to one another, making any analysis meaningless.

Overall, the positive correlation between $r$ and $\sigma$ indicates that, although the real systems are not constructed based on the subgraphs or more complex network structures, these systems show a solid dependency between the robustness and such structures. This indicates that the mechanisms that provide the robustness in biological systems might be similar to those in industrial systems, but the foundation of this mechanism is yet to be understood.

Although the process of creating product subnetworks required several assumptions to apply the model, this experiment is encouraging as it shows that it is possible to view the real-world networks through the prism of the model. However, to gain a more meaningful insight on an operational level about the explored systems the model needs to be further developed, simulating setups closer to the practical networks.

## Conclusion

Here we have presented a minimal model of supply networks. Although the model is based on one simple mechanism of matching the demand and supply, it proves to be powerful in describing the concepts of robustness and efficiency in supply networks. The optimal networks generated in this framework show structural patterns that are also typical to biological systems. This finding unites the nature of two network worlds—those found in natural systems that have been developed under evolutionary processes and industrial systems that are artificially created with the main goal of being cost-efficient. Having this evidence of similarity, it is possible to explore both systems from the perspective of another and potentially transfer the knowledge between them.

One of the questions addressed in our investigation is whether one can use motif patterns as a building recipe for robust networks. This approach turns out to be much more complex on the computational side, while also showing only a weak benefit in comparison to a random pattern. This numerical observation indicates that the family of networks with the given motif pattern is wider and includes not only the robust networks. An important finding, however, is that requiring a spatial locality of the network edges forces robust networks to adhere to the given motif signature. The discussed motif pattern thus should not be associated with robustness in isolation but should be augmented by some additional factors such as spatial aggregation.

Finally, while the suggested supply network model is minimal, it has substantial potential for further investigations. The most obvious approach is to go beyond the single-product dimension. In the explored setups there was only one producing node which, as can be seen from the industrial data, is usually not the case in reality. Also, the spatial distribution of the nodes is not uniform, especially in the case of worldwide supply networks. Another modification that might bring the model closer to the reality is a setup that has an overlay of different products, each with its own demanders and producers. In this setup, some transportation routes will be used to deliver multiple products, thus reducing the networks costs but imply a higher influence on the robustness. Alternatively, one can explore systems where the robustness has a tolerance margin. For example, networks that have at least $90\%$ demand satisfaction after damaging edges are still considered robust. In the single-product setup, reducing the demand satisfaction threshold results in simpler networks. However, combined with multi-product modification that should increase the complexity of the system the reduced demand satisfaction requirement would balance the system to be informative. Another possible approach is to apply different distance measures to compute network cost $c$. For example, instead of the standard Euclidean distance edges that are shorter than some threshold distance *x* can have a length of 1, while the longer edges can have an infinite length. This will model the situation when the delivery vehicles can travel no longer than *x* per one go.

With this minimal model, we hope to provide an interface between the multidisciplinary field of network science and research questions in supply network management.

## Supplementary Information

**Additional file 1**. Explanations of computational complexity of netowkrs metrics, as well as examples of 3-node subgraphs used in motif analysis, and figures with additional results.

## Data Availability

The source code of supply network model is available at the GitHub (https://github.com/ltvlx/Minimal-SN-model). Transportation data used in the “Application to industrial data” section are not publicly available due to the non-disclosure agreement with data provider.

## Footnotes

1 Note that the modular organization and community structure of complex networks is of course another mesoscale feature on a slightly larger level of organization, which revealed itself as highly relevant for the functional organization of complex networks (Newman 2006; Guimera and Amaral 2005; Hütt 2019). Note that one can also quantitatively study the interplay of these two mesoscales in complex networks (Fretter et al. 2012; Beber et al. 2012).
