# Supplementary material for: Local topological features of robust supply networks
Source: Appl Netw Sci. 2022 May 20;7(1):33. doi: 10.1007/s41109-022-00470-2 (PMC9122087; doi:10.1007/s41109-022-00470-2)
Supplement: Supplementary file 1 — Additional file 1. Explanations of computational complexity of netowkrs metrics, as well as examples of 3-node subgraphs used in motif analysis, and figures with additional results. [file 41109_2022_470_MOESM1_ESM.pdf]

Additional Files
Supplementary Information
Computational complexity
To compute the robustness, it is necessary to take every edge in the network and compute the demand satisfaction through all product paths. Computing the demand satisfaction takes $O(M)$, giving the total complexity of computing the robustness $O(M^2)$.
To compute network motifs, it is necessary to count all 3-node subgraphs in the original network, which takes $O(N^3)$. Then, the original network needs to be switch-randomized, which takes $O(M)$ operations, to make sure that every edge has a chance to be switched. The number of randomized networks to create distributions of subgraph counts is usually a constant independent of M or N. In our research, we work with networks that have $M \approx N$, which gives the total complexity of computing network motifs equal to $O(N^3M) \approx O(M^4)$.

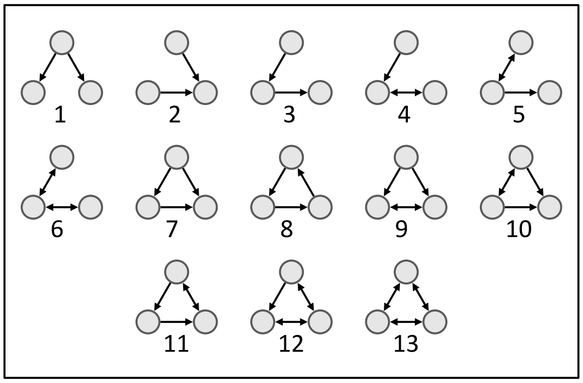

Figure S1: All 13 possible connected directed 3-node subgraphs for motif analysis.

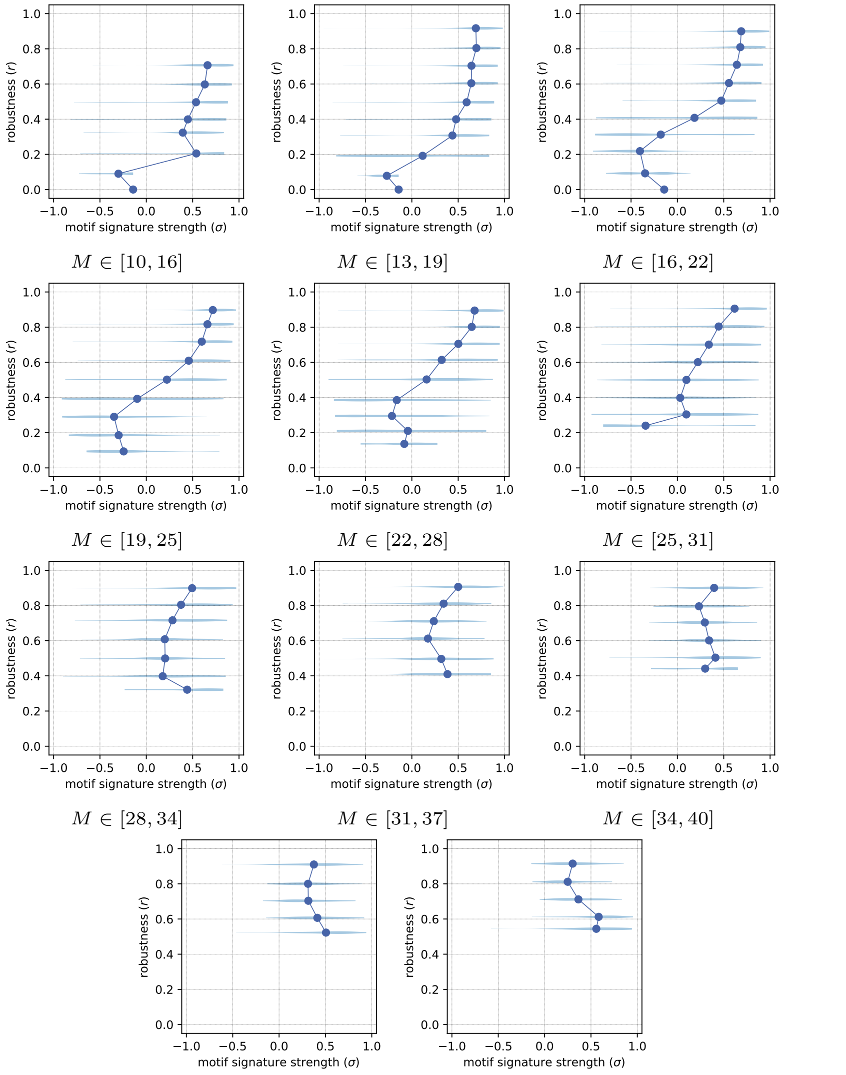

$M \in [10, 16]$ $M \in [13, 19]$ $M \in [16, 22]$

$M \in [19, 25]$ $M \in [22, 28]$ $M \in [25, 31]$

$M \in [28, 34]$ $M \in [31, 37]$ $M \in [34, 40]$

$M \in [37, 43]$ $M \in [40, 46]$

Figure S2: Distributions of σ values for different target r. Results from (c, r) optimization with $N = 20$.

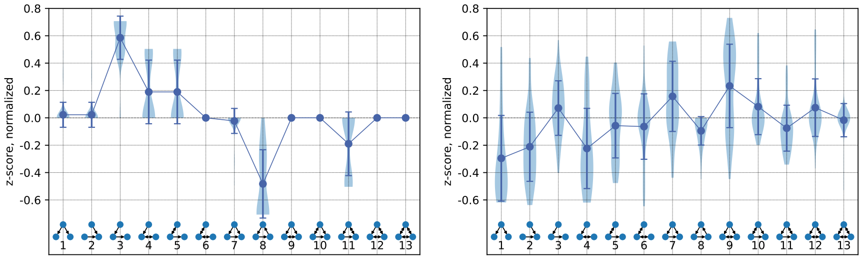

$M \in [8, 14]$, $r = 0.12$ $M \in [8, 14]$, $r = 0.92$

Figure S3: Motif patterns in (c, r) optimization with $N = 10$.

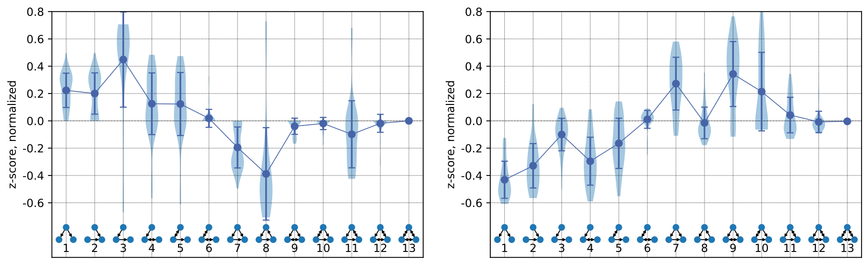

$M \in [22, 28]$, $r = 0.18$ $M \in [28, 34]$, $r = 0.97$

Figure S4: Motif patterns in (c, r) optimization with $N = 30$.

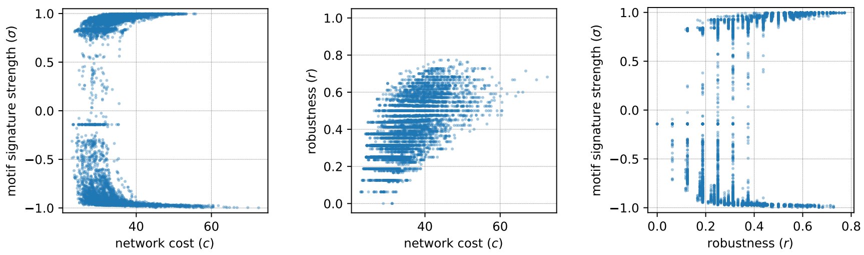

Figure S5: Pareto fronts in c, σ optimization with $N = 20$.

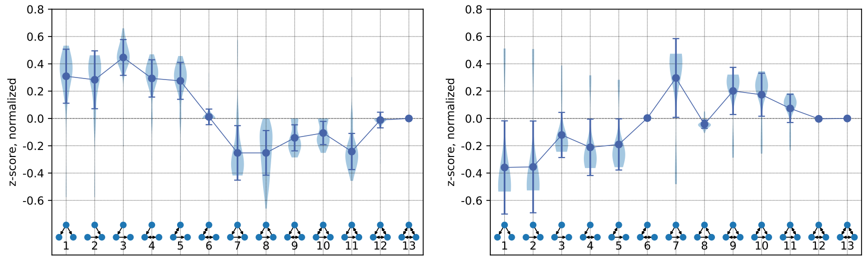

$M \in [16, 22], r = 0.19$ $M \in [16, 22], r = 0.60$

Figure S6: Motif patterns of networks with low and high robustness in c, σ optimization with $N = 20$.

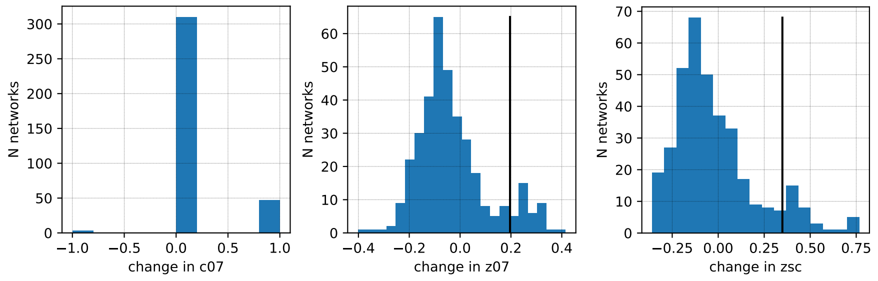

(a) FFL counts (b) FFL z-scores (c) σ

Figure S7: Histograms of changes in $c07$, $z07$, and σ values after edge insertion. After taking one base random network, extended networks are generated by taking the base network plus one additional edge that is not in the base network. For every possible extended network, $c07$, $z07$, and σ values are computed and the difference between these new values and the base network value is plotted. The x axis shows the change in $c07$, $z07$, and σ, while the y axis shows the number of extended networks that have this change. Extended networks with the highest change (above the black threshold line) are considered the best.

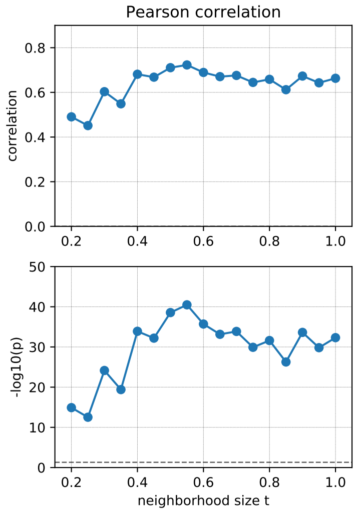

Figure S8: Pearson correlation and p-values between pattern strength and robustness for different neighborhood sizes t.

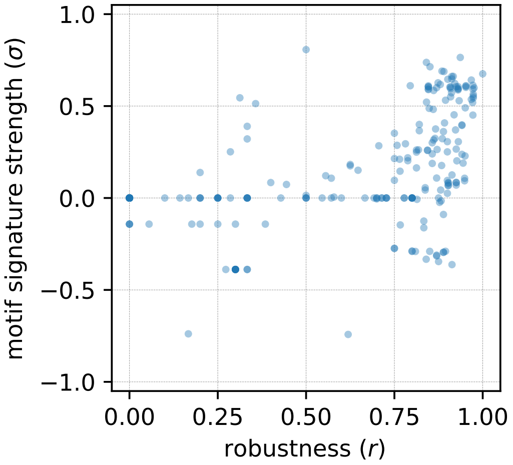

Figure S9: An example of product subnetworks from the real-world data in the r, σ parameter space with local neighborhood size parameter $t = 0.2$. This distribution of $(r,\ \sigma)$ values for the network is used to compute one point in the correlation curves (Fig. 10b and S8).
